# Hospital Urinary Tract Infections in Healthcare Units on the Example of Mazovian Specialist Hospital Ltd

**DOI:** 10.3389/fcimb.2022.891796

**Published:** 2022-07-11

**Authors:** Zuzanna Trześniewska-Ofiara, Mariola Mendrycka, Andrzej Cudo, Magdalena Szmulik, Agnieszka Woźniak-Kosek

**Affiliations:** ^1^ Department of Laboratory Diagnostics, Mazovian Specialist Hospital Ltd, Radom, Poland; ^2^ Department of Nursing, Faculty of Medical Sciences and Health Sciences, Kazimierz Pulaski University of Technology and Humanities, Radom, Poland; ^3^ Department of Experimental Psychology, Institute of Psychology, The John Paul II Catholic University of Lublin, Lublin, Poland; ^4^ Sysmex Poland Ltd, scientific aspect prepared in cooperation with Department of Laboratory Diagnostics, Military Institute of Medicine, Warsaw, Poland; ^5^ Department of Laboratory Diagnostics, Military Institute of Medicine, Warsaw, Poland

**Keywords:** urinary tract infections, microbiological diagnostics, nosocomial urinary tract infections, urine cultures, uropathogenic bacteria

## Abstract

Microbiological diagnostics is of great importance in limiting the spread of nosocomial infections. The information on etiological agents of infections and their susceptibility to antibiotics enables a quick response in the case of a suspected epidemic outbreak. The aim of this study is to analyze the incidence of nosocomial urinary tract infections among patients hospitalized in hospital wards over a period of 2 years and to determine the predominant etiological agent depending on the method of clinical specimen collection. Data from the Mazovian Specialist Hospital (MSH) in Radom constitute the material for the preparation of this study. Urine was collected using two methods. The first one was the method of collecting urine from the central stream, while the second method was urine collected from patients with a urinary catheter in place. The statistical calculations were conducted using the statistical software. Based on hospital data, it was shown that 5,870 urine tests were performed during the period under review, of which 2,070 were positive. The number of positive results in 2021 decreased by 2.84% compared to that in 2020. On the basis of the statistical analysis, differences in the occurrence of multiple strains were observed between catheter-based and midstream urine collection. Differences were observed especially for *Acinetobacter baumannii*, *Candida albicans*, *Escherichia coli*, and *Pseudomonas aeruginosa. A. baumannii*, *C. albicans*, and *P. aeruginosa* were significantly more frequently found in urine samples collected through the catheter than from the midstream. Furthermore, *E. coli* (51.56%) and *Enterococcus species* (25.46%) were more frequent when collected from the middle stream than when urine was collected through a catheter. However, for the strain *K. pneumoniae*, the results were comparable when urine was collected from catheterized patients (13.83%) and from midstream (13.35%). Urinary tract infection among hospitalized patients of the Mazovian Specialist Hospital in Radom was diagnosed quite frequently. In 2021, 32 more urine cultures were performed than in 2020. In the analyzed period, among all ordered urine cultures, 35.27% of samples were positive.

## Introduction

Nosocomial infections are a growing problem in the modern world. They affect all hospitals in the world (Khan et al., 2017; Tenney et al., 2018). According to the Polish legislation in force, a hospital infection is defined as any infection that occurred during the hospitalization of a patient but did not occur or remain in the incubation period during the admission of the patient to hospital (Wójkowska-Mach et al., 2013; World Health Organization, 2011).

Nosocomial infections affect both developed (7.0% of patients) and developing countries (10.0% of patients) (World Health Organization, 2011; Khan et al., 2017). In the United States, they affect 3.2% of hospitalized patients and 6.5% in the European Union (Sikora and Zahra, 2022). They can occur in different areas of healthcare services such as hospitals, long-term care facilities, and outpatient settings, and can also occur after a patient has been discharged from the hospital (World Health Organization, 2011; Sikora and Zahra, 2022).

Urinary tract infections in children and adults are one of the most common nosocomial (40.0%) and post-hospital infections (10.0%–20.0%). Approximately 15.0% of all antibiotics are prescribed for UI (Stamm and Norrby, 2001; Fihn, 2003; Bermingham and Ashe, 2012). Urinary tract infections are favored by anatomical defects, bladder dysfunction, bladder–urinary reflux, prolonged bladder catheterization, diagnostic and surgical procedures in the urogenital system, prostatic hypertrophy, diabetes mellitus, lithiasis, immunosuppression, pregnancy, and, to a great extent, sexual activity (Dellimore et al., 2013; Rowe and Juthani-Mehta, 2013; Hooton et al., 2020; Hsu and Melzer, 2018; Hatfield et al., 2018).

Urinary tract infections (UTI) may be acute, symptomatic infections of various courses and locations or may occur as asymptomatic bacteriuria. Very often, UTI is caused by bacteria that constitute the physiological flora of the gastrointestinal tract or microorganisms colonizing the skin (Gilbert et al., 2017). The main etiological agent of urinary tract infection is uropathogenic *Escherichia coli*, responsible for 80.0%–90.0% of community-acquired and 40% of hospital-acquired UI (Walters et al., 2012; Alfaresi et al., 2019). The development of urinary tract infection is usually by the ascending route (Kaper et al., 2004; Yu et al., 2020). The main symptoms are difficulty in urination, dysuria, hematuria, pyuria, frequent urination, nycturia, pain in the lumbar region, suprapubic region, bladder tenderness, fever, and foul smelling urine (Zalewska-Piątek et al., 2010; Schreiber et al., 2018).

Microbiological diagnostics is of great importance in limiting the spread of nosocomial infections. The information on etiological agents of infections and their susceptibility to antibiotics enables a quick response in the case of a suspected epidemic outbreak (Struelens et al., 2004; Trubiano and Padiglione, 2015; Klein and Hultgren, 2020).

The aim of this study was to analyze the incidence of nosocomial urinary tract infections among patients hospitalized in hospital wards over a period of 2 years and to determine the predominant etiological agent depending on the method of clinical specimen collection.

## Materials and Methods

Data from the Mazovian Specialist Hospital (MSH) in Radom (Poland) constitute the material for the preparation of this study. The hospital has 613 beds for patients, providing health services in 21 departments with subdivisions. The analysis covers data from the period January 1, 2020 to December 31, 2021. The material for the study was obtained from patients hospitalized in the analyzed period. Urine was collected using two methods. The first one was the method of collecting urine from the central stream, while the second method was urine collected from patients with a urinary catheter in place. Urine was not collected from patients by both methods simultaneously. These were patients hospitalized at MSH, and the choice of the method of collecting urine for examination was left to the discretion of the attending physician. The choice of urine collection method for culture was determined by the patient’s condition and the ability to collect sterile urine from the midstream. The urine, delivered to the Department of Laboratory Diagnostics, MSH, was cultured by the Hoeprich method on blood agar with 5% dehydrated sheep blood and MacConkey using 0.01 and 0.001 ml disposable plastic inoculation loop (10^2^ and 10^3^ dilutions). The culture was conducted under aerobic conditions at 35–37°C for 24–48 h. The result was presented as the number of grown microbial cells in 1 ml of urine. The pathogenic microorganisms were then identified and drug susceptibility determined using a Phoenix M50. Data were collected from 20 departments of MSH for analysis, i.e., Clinical Department of General Surgery, Oncology, Clinical Department of Neurology, Clinical Department of Oncology, Clinical Department of Rehabilitation, Clinical Department of Internal Medicine I, Clinical Department of Pediatrics, Clinical Department of Otolaryngology, Department of Trauma and Orthopedic Surgery, Department of Gynecology and Obstetrics, Department of Hematology, Department of Cardiology, Department of Pulmonology and Pulmonary Oncology, Department of Rheumatology, Internal Department II, Intensive Care Unit, Hospital Clinical Neurosurgery Ward, Department of Paediatric Surgery, Department of Neonatology, Department of Cardiac Surgery, and Department of Ophthalmology. The Hospital Emergency Ward was not included in the statistical analysis due to the short stay of patients in this ward. The criteria for the diagnosis of urinary tract infections were the clinical symptoms of the patients, which are often very different from asymptomatic URI to urosepsis. The clinical diagnosis was up to the attending physician of the patient hospitalized in MSH. In the patients studied, symptoms could be observed, e.g., dysuria, nycturia, tachyuria, and pyuria. On the other hand, the microbiological criteria for UTI were significant bakteriuria (≥10^4^ cfu/ml).

### Statistical Analysis

The χ^2^ test was used to assess the differences in positive urine tests results between the years analyzed (2020 and 2021). A similar test was used to verify the difference between catheter and midstream collection methods in the number of positive urine test detection for the whole hospital and selected hospital wards. Additionally, the χ^2^ test was used to assess the differences between the catheter and midstream collection method in the number of the most important pathogens (*Escherichia coli*, *Candida albicans*, *Klebsiella pneumoniae*, *Pseudomonas aeruginosa*, *Acinetobacter baumannii*, and *Enterococcus* spp.) found in the whole hospital and selected hospital wards. An analogous test was used to analyze the difference between the years analyzed (2020 and 2021) in the number of the most important pathogens found in selected hospital wards. The Yates’ correction (Yates, 1934) was used when expected cell frequencies were 5 or below in 2 × 2 contingency tables. The effect size for χ^2^ test was calculated using φ (Fritz et al., 2012) for variables with two categories and Cramér’s V (Cramér, 1961) for variables with more than two categories. The guidelines of Rea and Parker (1992) were used to interpret the effect sizes (φ and Cramér’s V): negligible (from 0.00 to 0.10), weak (from 0.10 to 0.20), moderate (from 0.20 to 0.40), relatively strong (from 0.40 to 0.60), strong (from 0.60 to 0.80), and very strong (from 0.80 to 1.00). In order to verify more precisely the differences between the analyzed variables when one of the variables had more than two categories, the adjusted standardized residuals were used (see Agresti, 2007). The absolute value of the adjusted standardized residuals >1.96 indicated a statistically significant result. The analyses used data on the number of urine tests performed regardless of the number of patients. The p-value considered statistically significant was 0.05.

The statistical calculations were conducted using the statistical software IBM SPSS version 27.

## Results

Based on hospital data, it was shown that 5,870 urine tests were performed during the period under review, of which 2,070 were positive. Positive results accounted for 35.26% of all urine tests. In 2020, 2,919 urine tests were performed for 31,653 hospitalizations. Urinalysis was performed for 9.22% of hospitalized patients. There were 1,071 positive results, and this accounted for 36.69% of all urine cultures ordered. For hospitalizations in 2021, 32,721 urine cultures were ordered for 9.02% of hospitalizations. In that year, 2,951 urine cultures were performed, of which 999 tests were positive. This represented 33.85% of all urine cultures ordered in 2021. The number of positive results in 2021 decreased by 2.84% compared to 2020.

The total number of midstream urine tests over 2 years was 3,882 samples, of which 1,434 were positive. This accounted for 36.94% of the midstream urine tests. On the other hand, the number of urine specimens collected from patients with a urinary catheter was 1,988, with 636 positive results. This represented 31.99% of urine specimens collected from patients with a urinary catheter. Among the midstream urine cultures, the number of positive tests decreased by 4.5% in 2021 compared to 2020, whereas if we consider urine from catheterized patients, the number of positive results decreased by only 0.5% in 2021 compared to 2020.

Nosocomial infections were found in patients who developed urinary tract infection after 72 h of admission of the patient to the hospital ward. Hospital-acquired infections among urinary tract infections in 2020 were 484, which accounted for 16.58% of the tests performed. Hospital-acquired infections among midstream urine tests were 264 or 54.55% of all infections. In contrast, nosocomial infections involving patients with urinary catheter accounted for 45.45%. In 2021, the number of nosocomial infections decreased by 1.53% from 2020 and was 444. Among midstream urine tests, infections occurring 72 h after patient admission were 255 (57.43%), while among urine samples from catheterized patients, positive results after 72 h were 489 (42.57%).

If we take into account the number of positive results in individual departments, we can see that in most of them, the number of positive results decreased in 2021 compared to 2020. Only the Department of Pulmonology and Pulmonary Oncology and Clinical Internal Medicine I showed an increase in the number of positive results in 2021 compared to 2020 (by 12.60% and 1.00%, respectively). In the case of the Pulmonology and Pulmonary Oncology Department, this may have been related to a 13.00% increase in the number of hospitalizations in 2021 relative to 2020. Due to the low number of urine culture tests ordered in the Departments of Pediatric Surgery, Neonatology, Ophthalmology, and Otolaryngology, these data are not relevant to this study (see **Table 1**).

**Table 1:** Number of positive urine results for individual hospital wards in 2020 and 2021.

Hospital wards	Percentage of positive urine culture results	
	2020	2021
Department of Pediatric Surgery	0.00%	33.30%
Clinical Oncology Department	10.70%	11.60%
Department of Neonatology	14.30%	25.00%
Department of Pulmonology and Pulmonary Oncology	15.10%	27.70%
Department of Ophthalmology	25.00%	33.30%
Department of Gynecology and Obstetrics	27.80%	23.60%
Intensive Care Unit	28.10%	24.60%
Department of Cardiac Surgery	31.80%	6.70%
Department of Rheumatology	34.10%	27.70%
Clinical Department of Otolaryngology	37.50%	0.00%
Clinical Department of Internal Medicine I	38.80%	39.80%
Clinical Department of Neurology	41.40%	38.40%
Internal Department II	41.40%	37.50%
Department of Cardiology	41.80%	25.40%
Department of Hematology	42.80%	34.30%
Clinical Department of General and Oncological Surgery	43.60%	36.10%
Clinical Department of Pediatrics	46.80%	36.90%
Department of Trauma and Orthopaedic Surgery	51.30%	43.30%
Hospital Clinical Department of Neurosurgery	52.60%	48.50%
Department of Rehabilitation	81.50%	67.60%

There was the difference between 2020 and 2021 year inthe frequencyof positive urine test (χ^2^_(df = 1)_ = 5.17, p = 0.023, φ = 0.030) and form the middle stream (χ^2^_(df = 1)_ = 8.51, p = 0.004, φ = 0.047) in the frequency of positive urine tests (see **Table 2**). Additionally, there was the difference between analysed time periods in the type of method used to collect the urine (χ^2^_(df = 1)_ = 27.12, p < 0.001, φ = 0.068). It should be noted that although the differences were statistically significant, the effect size was negligible (see Rea and Parker, 1992; Lin et al., 2013). The findings presented that there were differences between the methods of urine collection (from the catheter and from the middle stream) in the general frequency of positive urine tests [χ^2^_(df = 1)_ = 14.10, p = 0.001, φ = 0.049] and the frequency of positive urine tests among hospital infection [χ^2^_(df = 1)_ = 140.82, p = 0.001, φ = 0.261]. It should be noted that the effect size for the general frequency of positive urine tests was negligible, while that for the frequency of positive urine tests among hospital infections was moderate. Consequently, it may be pointed out that there were more positive urine tests from the catheter than from the middle stream among hospital infections (see **Table 3**).

**Table 2:** Numbers and percentage of urine tests in 2020 and 2021.

Variable	Category	Years				χ^2^	p	φ
		2020		2021				
		N	Percent	N	Percent			
Numbers of urine tests	Negative result	1,848	63.31%	1952	66.15%	5.17	0.023	0.030
	Positive result	1,071	36.69%	999	33.85%			
	Total	2,919	100.00%	2951	100.00%			
Numbers of urine tests	From the catheter	1,083	37.10%	905	30.67%	27.12	0.001	0.068
	From the middle stream	1,836	62.90%	2046	69.33%			
	Total	2,919	100.00%	2951	100.00%			
Numbers of urine tests from catheter	Negative result	734	67.77%	618	68.29%	0.06	0.807	0.005
	Positive result	349	32.23%	287	31.71%			
	Total	1,083	100.00%	905	100.00%			
Numbers of urine tests from the middle stream	Negative result	1,114	60.68%	1334	65.20%	8.51	0.004	0.047
	Positive result	722	39.32%	712	34.80%			
	Total	1,836	100.00%	2046	100.00%			
Numbers of hospital infections	From the catheter	220	45.45%	189	42.57%	0.78	0.376	0.029
	From the middle stream	264	54.55%	255	57.43%			
	Total	484	100.00%	444	100.00%			
Numbers of hospital infections from catheter *	Negative result	129	36.96%	98	34.15%	0.54	0.461	0.029
	Positive result	220	63.04%	189	65.85%			
	Total	349	100.00%	287	100.00%			
Numbers of hospital infections from the middle stream *	Negative result	458	63.43%	457	64.19%	0.09	0.767	0.008
	Positive result	264	36.57%	255	35.81%			
	Total	722	100.00%	712	100.00%			

*The number of hospital infections was referenced to the number of positive urine culture tests for each urine collection method.

**Table 3:** Urine test results in different urine collection methods.

Variable	Category	The method of urine collection				χ^2^	p	φ
		From the catheter		From the middle stream				
		N	Percent	N	Percent			
Numbers of urine tests	Negative result	1,352	68.01%	2448	63.06%	14.10	0.001	0.049
	Positive result	636	31.99%	1434	36.94%			
	Total	1,988	100.00%	3882	100.00%			
Numbers of hospital infections*	Negative result	227	35.69%	915	63.81%	140.82	0.001	0.261
	Positive result	409	64.31%	519	36.19%			
	Total	636	100.00%	1434	100.00%			

*The number of hospital infections was referenced to the number of positive urine culture tests for each urine collection method.

In the case of the analysis of the difference between the catheter and midstream collection methods in the number of positive urine test detection among selected hospital wards, there were statistically significant differences in only one ward: Internal Department II [χ^2^_(df = 1)_ = 10.70, p = 0.001, φ = 0.087]. More specifically, the positive urine test was more frequent when collected from the catheter than from the middle stream. The effect size was negligible. The detailed results are shown in **Supplementary Table SA**. It should be noted that the data from the following hospital wards were not analyzed due to insufficient sampling: Department of Gynecology and Obstetrics, Department of Ophthalmology, and Department of Pediatric Surgery.

In the case of the analysis of the difference between the catheter and midstream collection methods in the number of positive urine test detection after 72 h of hospitalization (hospital infection) among selected hospital wards, there were statistically significant differences in only one ward: Internal Department II [χ^2^_(df = 1)_ = 11.13, p < 0.001, φ = 0.176]. More precisely, in Internal Department II, the positive urine test after 72 h hospitalization (hospital infection) was more frequent when collected from the catheter than when collected from the middle stream. The effect size was weak. The detailed results are shown in **Supplementary Table SB**. It should be noted that the data from the following hospital wards were not analyzed due to insufficient sampling: Department of Gynecology and Obstetrics, Department of Neonatology, Department of Ophthalmology, and Department of Pediatric Surgery.

The results showed a difference between the urine test methods (catheter collection and middle stream collection) used to detect selected pathogens (*E. coli*, *C. albicans*, *K. pneumoniae*, *P. aeruginosa*, *A. baumannii*, and *Enterococcus* species) in the frequency of occurrence of the most important types of bacteria [χ^2^_(df =5)_ = 135.28, p < 0.001, Cramér’s V = 0.269]. The effect size was moderate. Considering the adjusted standardized residual values, differences between catheter collection and middle stream collection method were observed for *A. baumannii* (z = 4.5, p < 0.001), *C. albicans* (z = 8.4, p < 0.001), *E. coli* (z = 6.7, p < 0.001), and *P. aeruginosa* (z = 5.0, p < 0.001). Specifically, the presence of *A. baumannii*, *C. albicans*, and *P. aeruginosa* was more frequent with catheter collection than with middle stream collection method. In contrast, the *E. coli* was more frequent with middle stream collection than with catheter collection method. The detailed results are shown in **Table 4**. There was a difference between the urine test methods (catheter collection and middle stream collection) used to detect hospital infection in the frequency of occurrence of the most important types of bacteria [χ^2^_(df = 5)_ = 49.28, p < 0.001, Cramér’s V = 0.238]. The effect size was moderate. Taking into account the adjusted standardized residual values, differences between catheter collection and middle stream collection method were observed for *C. albicans* (z = 5.4, p < 0.001), *E. coli* (z = 4.3, p < 0.001), and *K. pneumoniae* (z = 2.1, p = 0.021). More precisely, *C. albicans* was more frequent with catheter collection than with middle stream collection method. However, *E. coli* and *K. pneumoniae* were more frequent with middle stream collection than with the catheter collection method. The detailed results are shown in **Table 4**.

**Table 4:** Presence of the most important types of pathogens.

Microorganisms	Number of pathogens found				χ^2^	p	Cramér’s V
	Catheter collection		Middle stream collection				
	N	Percent	N	Percent			
*Acinetobacter baumannii*	31	6.13%	28	2.05%	135.28	0.001	0.269
*Candida albicans*	76	15.02%	53	3.89%			
*Enterococcus species*	109	21.54%	347	25.46%			
*Escherichia coli*	173	34.19%	704	51.65%			
*Klebsiella pneumoniae spp pneumoniae*	70	13.83%	182	13.35%			
*Pseudomonas aeruginosa*	47	9.29%	49	3.60%			
Total	506	100.00%	1363	100.00%			
**Microorganisms**	**Hospital infections**						
	**Catheter collection**		**Middle stream collection**				
	**N**	**Percent**	**N**	**Percent**			
*Acinetobacter baumannii*	28	7.89%	25	4.84%	49.28	0.001	0.238
*Candida albicans*	66	18.59%	35	6.77%			
*Enterococcus species*	133	37.46%	197	38.10%			
*Escherichia coli*	59	16.62%	151	29.21%			
*Klebsiella pneumoniae spp pneumoniae*	37	10.42%	79	15.28%			
*Pseudomonas aeruginosa*	32	9.01%	30	5.80%			
Total	355	100.00%	517	100.00%			

There was a difference between the years 2020 and 2021 in terms of the presence of the most important types of pathogens on selected hospital wards [χ^2^_(df = 19)_ = 95.03, p < 0.001, Cramér’s V = 0.221]. The effect size was moderate. Taking into account the adjusted standardized residual values, differences between 2020 and 2021 were observed for Cardiology Department (z = 2.0, p = 0.046), Department of Pulmonology and Pulmonary Oncology (z = 6.6, p < 0.001), Internal Department II (z = 3.0, p = 0.003), and Intensive Care Unit (z = 4.8, p < 0.001). More precisely, in the hospital wards, such as Department of Pulmonology and Pulmonary Oncology and Internal Department II, the most important types of pathogens were fewer in 2020 than in 2021. Additionally, in hospital wards, such as the Cardiology Department and Intensive Care Unit, more major types of pathogens were found in 2020 than in 2021. The results showed a difference between 2020 and 2021 in terms of the presence of the most important types of pathogens in hospital infections in selected hospital wards [χ^2^_(df = 17)_ = 68.45, p < 0.001, Cramér’s V = 0.280]. The effect size was moderate. Considering the adjusted standardized residual values, differences between 2020 and 2021 were observed for Clinical Department of Pediatrics (z = 2.3, p = 0.022), Department of Pulmonology and Pulmonary Oncology (z = 5.8, p < 0.001), Internal Department II (z = 2.7, p = 0.007), and Intensive Care Unit (z = 4.0, p < 0.001). Specifically, in hospital wards, such as the Department of Pulmonology and Pulmonary Oncology and Internal Department II, the most important types of pathogens in hospital infections were fewer in 2020 than in 2021. Additionally, in hospital wards, such as the Clinical Department of Pediatrics and Intensive Care Unit, the most important types of pathogens in hospital infections were more in 2020 than in 2021. The detailed results are shown in **Table 5**.

**Table 5:** Presence of the most common types of pathogens on selected hospital wards.

Number of the most important types of pathogens—total							
Hospital wards	Years				χ^2^	p	Cramér’s V
	2020		2021				
	N	Percent	N	Percent			
Clinical Department of General and Oncological Surgery	16	1.57%	13	1.39%	95.03	0.001	0.221
Clinical Department of Neurology	53	5.21%	57	6.11%			
Clinical Department of Oncology	53	5.21%	64	6.86%			
Clinical Department of Otolaryngology	4	0.39%	0	0.00%			
Clinical Department of Pediatrics	83	8.15%	60	6.43%			
Clinical Department of Rehabilitation	26	2.55%	24	2.57%			
Clinical Department of Internal Medicine I	115	11.30%	85	9.11%			
Department of Pediatric Surgery	0	0.00%	2	0.21%			
Department of Trauma and Orthopaedic Surgery	19	1.87%	28	3.00%			
Department of Gynecology and Obstetrics	19	1.87%	12	1.29%			
Department of Hematology	66	6.48%	43	4.61%			
Department of Cardiac Surgery	5	0.49%	2	0.21%			
Department of Cardiology	33	3.24%	17	1.82%			
Department of Neonatology	1	0.10%	1	0.11%			
Department of Ophthalmology	1	0.10%	1	0.11%			
Department of Pulmonology and Pulmonary Oncology	10	0.98%	62	6.65%			
Department of Rheumatology	26	2.55%	25	2.68%			
Internal Department II	251	24.66%	286	30.65%			
Intensive Care Unit	216	21.22%	121	12.97%			
Hospital Clinical Department of Neurosurgery	21	2.06%	30	3.22%			
Total	1,018	100.00%	933	100.00%			
**Number of the most important types of pathogens in hospital infections**							
**Hospital wards**	**Years**				**χ^2^**	**p**	**Cramér’s V**
	**2020**		**2021**				
	**N**	**Percent**	**N**	**Percent**			
Clinical Department of General and Oncological Surgery	16	3.49%	9	2.16%	68.45	0.001	0.280
Clinical Department of Neurology	29	6.32%	34	8.17%			
Clinical Department of Oncology	23	5.01%	27	6.49%			
Clinical Department of Otolaryngology	4	0.87%	0	0.00%			
Clinical Department of Pediatrics	6	1.31%	0	0.00%			
Clinical Department of Rehabilitation	25	5.45%	20	4.81%			
Clinical Department of Internal Medicine I	37	8.06%	31	7.45%			
Department of Trauma and Orthopaedic Surgery	18	3.92%	18	4.33%			
Department of Gynecology and obstetrics	1	0.22%	1	0.24%			
Department of Hematology	26	5.66%	17	4.09%			
Department of Cardiac Surgery	4	0.87%	2	0.48%			
Department of Cardiology	23	5.01%	15	3.61%			
Department of Ophthalmology	1	0.22%	0	0.00%			
Department of Pulmonology and Pulmonary Oncology	6	1.31%	43	10.34%			
Department of Rheumatology	8	1.74%	4	0.96%			
Internal Department II	52	11.33%	74	17.79%			
Intensive Care Unit	159	34.64%	93	22.36%			
Hospital Clinical Department of Neurosurgery	21	4.58%	28	6.73%			
Total	459	100.00%	416	100.00%			

The findings showed a difference between urine test methods (catheter collection and middle stream collection) in the frequency of occurrence of the most important pathogens (*E. coli*, *C. albicans*, *K. pneumoniae*, *P. aeruginosa*, *A. baumannii*, and *Enterococcus* species) on selected hospital wards [χ^2^_(df =19)_ = 908.42, p < 0.001, Cramér’s V = 0.682]. The effect size was strong. Considering the adjusted standardized residual values, differences between catheter collection and middle stream collection method were observed for Clinical Department of Neurology (z = 4.1, p < 0.001), Clinical Department of Oncology (z = 5.1, p < 0.001), Clinical Department of Pediatrics (z = 7.9, p < 0.001), Clinical Department of Internal Medicine I (z = 6.0, p < 0.001), Department of Trauma and Orthopaedic Surgery(z = 4.5, p < 0.001), Department of Gynecology and Obstetrics (z = 3.7, p < 0.001), Department of Hematology (z = 6.0, p < 0.001), Department of Cardiology (z = 4.7, p < 0.001), Department of Pulmonology and Pulmonary Oncology (z = 5.7, p < 0.001), Department of Rheumatology (z = 3.8, p < 0.001), Intensive Care Unit (z = 27.3, p < 0.001), and Hospital Clinical Department of Neurosurgery (z = 3.9, p < 0.001). More precisely, the presence of the most important pathogens was more frequent with catheter collection than with middle stream collection method for the Intensive Care Unit and Hospital Clinical Department of Neurosurgery. In contrast, the presence of the most important pathogens was less frequent with catheter collection than with middle stream collection method for the Clinical Department of Neurology, Clinical Department of Oncology, Clinical Department of Pediatrics, Clinical Department of Internal Medicine I, Department of Trauma and Orthopaedic Surgery, Department of Gynecology and Obstetrics, Department of Hematology, Department of Cardiology, Department of Pulmonology and Pulmonary Oncology, and Department of Rheumatology. The detailed results are shown in **Table 6**. There was a difference between urine test methods (catheter collection and middle stream collection) in the frequency of occurrence of the most important pathogens (*E. coli*, *C. albicans*, *K. pneumoniae*, *P. aeruginosa*, *A. baumannii*, and *Enterococcus* species) in hospital infections in selected hospital wards [χ^2^_(df = 18)_ = 758.45, p < 0.001, Cramér’s V = 0.764]. The effect size was strong. Taking into account the adjusted standardized residual values, differences between catheter collection and middle stream collection method were observed for Clinical Department of Neurology (z = 3.8, p < 0.001), Clinical Department of Oncology (z = 3.7, p < 0.001), Clinical Department of Pediatrics (z = 5.8, p < 0.001), Clinical Department of Internal Medicine I (z = 6.5, p < 0.001), Department of Trauma and Orthopaedic Surgery (z = 3.8, p < 0.001), Department of Gynecology and Obstetrics (z = 2.8, p = 0.005), Department of Hematology (z = 5.0, p < 0.001), Cardiology Department (z = 4.3, p < 0.001), Department of Pulmonology and Pulmonary Oncology (z = 4.6, p < 0.001), Department of Rheumatology (z = 2.9, p = 0.004), Internal Department II (z = 3.5, p < 0.001), Intensive Care Unit (z = 25.7, p < 0.001), and Hospital Clinical Department of Neurosurgery (z = 4.8, p < 0.001). More precisely, the presence of the most important pathogens in hospital infections was more frequent with catheter collection than with middle stream collection method for Intensive Care Unit and Hospital Clinical Department of Neurosurgery. In contrast, the presence of the most important pathogens was less frequent with catheter collection than with middle stream collection method for Clinical Department of Neurology, Clinical Department of Oncology, Clinical Department of Pediatrics, Clinical Department of Internal Medicine I, Department of Trauma and Orthopaedic Surgery, Department of Gynecology and Obstetrics, Department of Hematology, Cardiology Department, Department of Pulmonology and Pulmonary Oncology, Department of Rheumatology, and Internal Department II. The detailed results are shown in **Table 6**.

**Table 6:** Presence of the most common types of pathogens on selected hospital wards.

Number of the most important types of pathogens—total							
Hospital wards	The method of urine collection				χ^2^	p	Cramér’s V
	From the catheter		From the middle stream				
	N	Percent	N	Percent			
Clinical Department of General and Oncological Surgery	4	0.68%	25	1.83%	908.42	0.001	0.682
Clinical Department of Neurology	14	2.39%	96	7.03%			
Clinical Department of Oncology	10	1.71%	107	7.83%			
Clinical Department of Otolaryngology	0	0.00%	4	0.29%			
Clinical Department of Pediatrics	1	0.17%	142	10.40%			
Clinical Department of Rehabilitation	16	2.74%	34	2.49%			
Clinical Department of Internal Medicine I	23	3.93%	177	12.96%			
Department of Pediatric Surgery	0	0.00%	2	0.15%			
Department of Trauma and Orthopaedic Surgery	0	0.00%	47	3.44%			
Department of Gynecology and obstetrics	0	0.00%	31	2.27%			
Department of Hematology	5	0.85%	104	7.61%			
Department of Cardiac Surgery	4	0.68%	3	0.22%			
Department of Cardiology	0	0.00%	50	3.66%			
Department of Neonatology	0	0.00%	2	0.15%			
Department of Ophthalmology	0	0.00%	2	0.15%			
Department of Pulmonology and Pulmonary Oncology	0	0.00%	72	5.27%			
Department of Rheumatology	3	0.51%	48	3.51%			
Internal Department II	167	28.55%	370	27.09%			
Intensive Care Unit	310	52.99%	27	1.98%			
Hospital Clinical Department of Neurosurgery	28	4.79%	23	1.68%			
Total	585	100.00%	1366	100.00%			
**Number of the most important types of pathogens in hospital infections**							
**Hospital wards**	**The method of urine collection**				**χ^2^**	**p**	**Cramér’s V**
	**From the catheter**		**From the middle stream**				
	**N**	**Percent**	**N**	**Percent**			
Clinical Department of General and Oncological Surgery	3	0.8%	22	2.3%	758.45	0.001	0.764
Clinical Department of Neurology	8	2.3%	77	8.2%			
Clinical Department of Oncology	7	2.0%	70	7.4%			
Clinical Department of Otolaryngology	0	0.0%	4	0.4%			
Clinical Department of Pediatrics	0	0.0%	83	8.8%			
Clinical Department of Rehabilitation	14	3.9%	32	3.4%			
Clinical Department of Internal Medicine I	5	1.4%	130	13.8%			
Department of Trauma and Orthopaedic Surgery	0	0.0%	37	3.9%			
Gynecology and obstetrics Department	0	0.0%	20	2.1%			
Department of Hematology	3	0.8%	80	8.5%			
Department of Cardiac Surgery	4	1.1%	3	0.3%			
Department of Cardiology	0	0.0%	48	5.1%			
Department of Neonatology	0	0.0%	1	0.1%			
Department of Ophthalmology	0	0.0%	1	0.1%			
Department of Pulmonology and Pulmonary Oncology	0	0.0%	53	5.6%			
Department of Rheumatology	1	0.3%	27	2.9%			
Internal Department II	50	14.1%	215	22.8%			
Intensive Care Unit	232	65.4%	20	2.1%			
Hospital Clinical Department of Neurosurgery	28	7.9%	21	2.2%			
Total	355	100.0%	944	100.0%			

## Discussion

Medicine today uses many devices to assist patients in their recovery; unfortunately, these devices can become the cause of healthcare-associated infections. These include ventilators or catheters (Weiner et al., 2016). Among ICU patients, in addition to mechanical ventilation, the use of invasive medical procedures and very often reduced immunity pose a risk (Khan et al., 2015). Healthcare-associated infections include respiratory pneumonia, bloodstream infection associated with a vascular line, and urinary tract infection associated with a urinary catheter (Edwardson and Cairns, 2019).

Urinary tract infection among hospitalized patients of the Mazovian Specialist Hospital in Radom was diagnosed quite frequently (**Table 2**). In 2021, 32 more urine cultures were performed than in 2020, but the number of positive results in 2021 was lower by 2.84% compared to that in 2020. In the analyzed period, among all ordered urine cultures, 35.27% of the samples were positive.

Hospital infections are not only a problem in Poland. A similar result was obtained in China, where, during hospitalization of patients, the most common infections were urinary tract infections, accounting for 38.1% of all other infections. On the other hand, respiratory tract infections (26.8%) and bloodstream infections (12.5%) also occurred but less frequently (Jiang et al., 2020). According to Remschmidt et al. (2018) in Germany, there has been an increase from 2.9% to 9.9% in urinary tract infections caused by *Enterococcus* in hospital patients in recent years, which confirms the existing problem of nosocomial infections caused by uropathogens. Studies conducted by Su et al. (2021) in China in 2019 and 2020 on hospital-acquired infections in general indicated a reduction in the rate of critical and fatal cases. This may have been due to increased rates of proper hand washing and disinfection, use of more gloves and protective aprons, among other factors. During the period analyzed by these researchers, the frequency of nosocomial respiratory and gastrointestinal infections decreased in most wards except the Intensive Care Unit. In contrast, no significant differences were found when comparing catheter-related nosocomial infections.

Based on the study, it can be indicated that there were more positive urine test results collected from catheter than from midstream among the nosocomial infections. Statistically significant differences in the number of positive urine test results collected by catheter versus midstream method occurred only in one case from the Internal Department II. Furthermore, a positive urine result after 72 h of a patient’s hospitalization in the Internal Department II was also more frequent with catheter-based collection than with midstream collection.

Urinary-catheter-related infection is one of the more common nosocomial infections and has been studied by many authors (Foxman, 2002; Flores-Mireles et al., 2015; Eliakim-Raz et al., 2019; Garcia-Bustos et al., 2021). Infections affect both the upper and lower urinary tract (Gharbi et al., 2019). The severity of these infections is determined, among other things, by the ability of the bacteria to adhere to the epithelium lining in the urinary tract and the ability to form a biofilm, particularly in catheters inserted into the bladder. It is important to remember that biofilm formation begins immediately after insertion of the catheter into the bladder (Schulze et al., 2021).

On the basis of statistical analysis, differences in the occurrence of multiple strains were observed between catheter-based and midstream urine collection (**Table 4**). Differences were observed especially for *A. baumannii* (z = 4.3, p < 0.001), *C. albicans* (z = 8.1, p < 0.001), *E. coli* (z = 9.0, p < 0.001), and *P. aeruginosa* (z = 4.7, p < 0.001). *A. baumannii*, *C. albicans*, and *P. aeruginosa* were significantly more frequently found in urine samples collected through the catheter than from the midstream. Furthermore, *E. coli* (51.56%) and *Enterococcus* species (25.46%) were more frequent when collected from the middle stream than when urine was collected through a catheter. However, for the strain *K. pneumoniae*, the results were comparable when urine was collected from catheterized patients (13.83%) and from midstream (13.35%).

Gram-negative bacillus *K. pneumoniae* as an etiological agent of UTI is of significant importance in the therapeutic and epidemiological aspects of the hospital. The increasing resistance of *K. pneumoniae* to antibiotics is a problem not only for MSH in Radom but also for other hospitals in Poland and abroad (Clegg and Murphy, 2016; Lee et al., 2017; Mazzariol et al., 2017)). Non-fermenting bacilli *P. aeruginosa* and *A. bauamnnii* are responsible for urinary tract infections most frequently in patients hospitalized in (ICU). These were patients often subjected to broad-spectrum antibiotic therapy, hospitalized for a long time, and with reduced immunity. These factors significantly favor nosocomial infections caused by these microorganisms (Jain and Danziger, 2004; Michalopoulos and Falagas, 2010; Paz-Zarza et al., 2019).

Moreover, Gram-positive granules of the genus *Enterococcus* spp. are a significant microorganism causing urinary tract infections (O’brien et al., 2016; Tamadonfar et al., 2019). According to the literature reports, *E. faecalis* is the common etiological agent of UTI. In our study (see **Table 4**), *Enterococcus* species was isolated from urine samples collected through a catheter (21.54%) and from the midstream (25.46%), which confirmed the reports of other authors that this bacterium is also a common agent of urinary tract infections. However, the predominant microorganism in urinary tract infections during the analyzed period was Gram-negative *E. coli*, which occurred more frequently when urine was collected from the midstream (51.65%) than through a catheter (34.19%) (**Table 4**). When comparing the frequency of this pathogen with other microorganisms in the urine samples collected from patients, this bacterium was diagnosed most frequently in both catheter and midstream urine. This confirms reports by other authors that *E. coli* is the most common etiological agent of UAS in many countries (Robino et al., 2014; da Cruz Campos, 2020; Serretiello et al., 2021). The ease of causing urinary tract infections by this microorganism is due to the many virulence factors that it possesses. *E. coli* produce many adhesins and invasins that enable bacteria to adhere to host cells. They also produce many toxins that modulate the immune response and siderophores necessary for iron uptake (Frick-Cheng et al., 2020; Bunduki et al., 2021).

When analyzing the type of microorganisms present in urine samples in individual hospital wards, fewer major types of pathogens were found in 2020 than in 2021 in the Pulmonology and Pulmonary Oncology Unit and the Internal Medicine II Unit. In addition, more major types of pathogens were found in 2020 than in 2021 in hospital wards such as the Cardiology Department and the ICU (**Table 6**).

The results showed a statistically significant difference between 2020 and 2021 in terms of the presence of the most important types of pathogens in nosocomial infections in selected hospital wards.

In the case of the ICU and the Hospital Neurosurgery Clinical Department, the presence of the most important pathogens was more frequent with catheter-based urine collection than with the medium-stream method. In contrast, the presence of major pathogens was less frequent when urine was collected by catheter than by midstream from patients in the Hematology Department, Cardiology Department, Pulmonology and Pulmonary Oncology Department, and Rheumatology Department (**Table 6**).

According to Vincent et al. (2009), patients admitted to intensive care, transplant, or neonatal units are at highest risk of nosocomial infections. The main causes of nosocomial infections are the easy transmission of pathogens from one patient to another and the colonization in the hospital environment (Khan et al., 2015). In addition to these are increase in antibiotic consumption, too long a patient stay in hospital, colonization with multi-drug resistant strains, and an aging population (Bereket et al., 2012).

Microorganisms have the ability to enter the urinary tract causing infections despite many of the body’s defense mechanisms (Seo et al., 2014). Factors protecting the urinary system from infection include mechanical flushing of microorganisms during micturition, the acid reaction of urine, the physiological flora of the urethral region inhibiting bacterial adhesion, and the bactericidal effect of mucopolysaccharides of the bladder mucosa (Schulze et al., 2021). However, the biggest problem is the catheterized patient. To prevent UTI in such patients, the they should be catheterized only when absolutely necessary (in patients requiring prolonged immobilization, those who need support in healing of perineal wounds, those with urinary incontinence, and those who underwent selected surgeries), the catheter should be kept as short as possible, the catheter should be inserted with disposable equipment using aseptic principles, the catheter-drain-tank system should be kept leakproof, and the care plan for the patient should be prepared (including increased fluid intake) (Klein and Hultgren, 2020). The duration of urinary catheter maintenance is determined by its patency, and monitoring this is the nurse’s responsibility. An important aspect concerning the medical staff is also the observance of the sanitary regime. All nursing procedures should be carried out with disposables, under aseptic conditions to prevent the transmission of pathogens among patients (Health Quality Ontario, 2019).

Another important element concerning urinary tract infections is rational antibiotic therapy. In cases of asymptomatic bacteriuria, antibiotics are not recommended, except for pregnancy and planned urological surgery. The use of infection prophylaxis in patients with a urinary catheter is a mistake (Köves et al., 2017; Saffar et al., 2008; Singha et al., 2017). Unfortunately, we still encounter this practice very often. Failure to adhere to current recommendations promotes increasing antimicrobial resistance among microorganisms, therapeutic difficulties, and the emergence of nosocomial infections (Nicolle et al., 2019).

## Conclusion

There was a statistically significant detect difference in the frequency of positive urine tests between 2020 and 2021 depending on the method of collection. Positive urinalysis was more frequent when collecting material from catheterized patients than from midstream. The same relationship was obtained for urine samples collected after 72 h of hospitalization where nosocomial uropathogen infections were more frequent in catheterized patients in the Internal Department II. The results were statistically significant only for this ward. In the urine samples examined, the presence of strains, namely, *A. baumannii*, *C. albicans*, and *P. aeruginosa*, were more frequent when urine was collected from the catheter than from the midstream. In contrast, *E. coli* and *Enterococcus* were more frequent when urine was collected from the midstream than through a catheter. There was a statistical difference in the presence of key pathogen types in selected hospital wards between 2020 and 2021. Differences between 2020 and 2021 were observed for the Department of Cardiology, the Department of Pulmonology and Pulmonary Oncology, the Department of Internal Medicine and the ICU. In hospital wards such as the Pulmonology and Pulmonary Oncology Unit and the Internal Department II, fewer major pathogen types were found in 2020 than in 2021. Additionally, in inpatient wards such as the Department of Cardiology and ICU, more major types of pathogens were found in 2020 than in 2021. A statistical difference was found in the method of urine testing (catheter collection and midstream collection) and the prevalence of the most important pathogens (*E. coli*, *C. albicans*, *K. pneumoniae*, *P. aeruginosa*, *A. baumannii*, *Enterococcus* species) in selected hospital wards. The presence of the most important pathogens was more frequent with catheter-based collection than with the medium-stream method in the case of the ICU and the Hospital Clinical Department of Neurosurgery. In contrast, the presence of major pathogens was less frequent with catheter-based than midstream urine collection in the Hematology Department, Cardiology Department, Pulmonology and Pulmonary Oncology Department, and Rheumatology Department.

## Data Availability Statement

The original contributions presented in the study are included in the article/**Supplementary Material**. Further inquiries can be directed to the corresponding authors.

## Author Contributions

The author contributions statement must describe the contributions of individual authors referred to by their initials and, in doing so, all authors agree to be accountable for the content of the work. ZT-O contributed to the conception and design of the manuscript and collection of the patient database and wrote the first draft of the manuscript. MM participated in the conception of the draft manuscript and contributed to the writing of the discussion and selection of the literature. AC contributed to the selection of the statistical methodology and statistical analysis of the results. MS contributed to the review and selection of the literature for the manuscript. AW-K contributed to the conception and design of the manuscript and made the final revision of the manuscript. All authors read and approved the submitted version of the paper.

## Conflict of Interest

Author MS is employed by Sysmex Poland Ltd. Warsaw and privately is Ph.D. reseacher at Department of Laboratory Diagnostics, Military Institute of Medicine, Warsaw, Poland.

The remaining authors declare that the research was conducted in the absence of any commercial or financial relationships that could be construed as a potential conflict of interest.

## Publisher’s Note

All claims expressed in this article are solely those of the authors and do not necessarily represent those of their affiliated organizations, or those of the publisher, the editors and the reviewers. Any product that may be evaluated in this article, or claim that may be made by its manufacturer, is not guaranteed or endorsed by the publisher.

## Abbreviations

MSH in Radom, Poland, Mazovian Specialist Hospital in Radom, Poland; UTI, urinary tract infections; ICU, intensive care unit.
