# Supplementary material for: Hospital Urinary Tract Infections in Healthcare Units on the Example of Mazovian Specialist Hospital Ltd
Source: Front Cell Infect Microbiol. 2022 Jul 11;12:891796. doi: 10.3389/fcimb.2022.891796 (PMC9309389; doi:10.3389/fcimb.2022.891796)
Supplement: Supplementary file 1 [file Table_1.pdf]

Table A. The urine test results in different urine collection methods among selected hospital wards.

Variable	Category	The method of urine collection				χ^2	p	φ
		From the catheter		From the middle stream				
		N	Percent	N	Percent			
Clinical Department of General and Oncological Surgery	Negative result	7	53.85%	38	61.29%	0.25	0.618	0.058
	Positive result	6	46.15%	24	38.71%			
	Total	13	100.00%	62	100.00%			
Clinical Department of Neurology	Negative result	23	58.97%	157	60.38%	0.03	0.867	0.010
	Positive result	16	41.03%	103	39.62%			
	Total	39	100.00%	260	100.00%			
Clinical Oncology Department	Negative result	13	61.90%	181	60.33%	0.02	0.887	0.008
	Positive result	8	38.10%	119	39.67%			
	Total	21	100.00%	300	100.00%			
Clinical Department of Otolaryngology	Negative result	3	100.00%	8	72.73%	0.05	0.821	0.273
	Positive result	0	0.00%	3	27.27%			
	Total	3	100.00%	11	100.00%			
Clinical Department of Pediatrics	Negative result	2	66.67%	202	57.71%	0.01	1.000	0.017
	Positive result	1	33.33%	148	42.29%			
	Total	3	100.00%	350	100.00%			
Clinical Department of Internal Medicine I	Negative result	29	60.42%	275	60.84%	0.01	0.954	0.003
	Positive result	19	39.58%	177	39.16%			
	Total	48	100.00%	452	100.00%			
Department of Trauma and Orthopaedic Surgery	Negative result	0	0.00%	57	54.29%	0.01	0.939	0.105
	Positive result	1	100.00%	48	45.71%			
	Total	1	100.00%	105	100.00%			
Department of Hematology	Negative result	10	62.50%	194	61.39%	0.01	0.929	0.005
	Positive result	6	37.50%	122	38.61%			
	Total	16	100.00%	316	100.00%			
Department of Cardiac Surgery	Negative result	12	80.00%	17	77.27%	0.01	1.000	0.033
	Positive result	3	20.00%	5	22.73%			
	Total	15	100.00%	22	100.00%			
Department of Cardiology	Negative result	1	100.00%	92	65.25%	0.01	1.000	0.061
	Positive result	0	0.00%	49	34.75%			
	Total	1	100.00%	141	100.00%			
Departmentof Neonatology	Negative result	5	100.00%	4	66.67%	0.41	0.521	0.430
	Positive result	0	0.00%	2	33.33%			
	Total	5	100.00%	6	100.00%			
Department of Pulmonology and Pulmonary Oncology	Negative result	2	100.00%	214	75.35%	0.01	1.000	0.048
	Positive result	0	0.00%	70	24.65%			
	Total	2	100.00%	284	100.00%			
Department of Rehabilitation	Negative result	5	26.32%	11	26.19%	0.01	0.992	0.001
	Positive result	14	73.68%	31	73.81%			
	Total	19	100.00%	42	100.00%			
Department of Rheumatology	Negative result	0	0.00%	117	70.06%	1.86	0.173	0.164
	Positive result	2	100.00%	50	29.94%			
	Total	2	100.00%	167	100.00%			
Internal Department II	Negative result	198	53.66%	668	63.32%	10.70	0.001	0.087
	Positive result	171	46.34%	387	36.68%			
	Total	369	100.00%	1055	100.00%			
Intensive Care Unit	Negative result	1016	73.89%	60	65.93%	2.77	0.096	0.043
	Positive result	359	26.11%	31	34.07%			
	Total	1375	100.00%	91	100.00%			
Hospital Clinical Department of Neurosurgery	Negative result	26	46.43%	27	54.00%	0.61	0.436	0.076
	Positive result	30	53.57%	23	46.00%			
	Total	56	100.00%	50	100.00%			
