# Supplementary material for: Hospital Urinary Tract Infections in Healthcare Units on the Example of Mazovian Specialist Hospital Ltd
Source: Front Cell Infect Microbiol. 2022 Jul 11;12:891796. doi: 10.3389/fcimb.2022.891796 (PMC9309389; doi:10.3389/fcimb.2022.891796)
Supplement: Supplementary file 2 [file Table_2.pdf]

Table B. The urine test results after 72 hours (hospital infection) in different urine collection methods among selected hospital wards.

Variable	Category	The method of urine collection				χ^2	p	φ
		From the catheter		From the middle stream				
		N	Percent	N	Percent			
Clinical Department of General and Oncological Surgery	Negative result	7	58.33%	27	56.25%	0.02	0.896	0.017
	Positive result	5	41.67%	21	43.75%			
	Total	12	100.00%	48	100.00%			
Clinical Department of Neurology	Negative result	12	54.55%	89	59.73%	0.21	0.644	0.035
	Positive result	10	45.45%	60	40.27%			
	Total	22	100.00%	149	100.00%			
Clinical Department of Oncology	Negative result	4	40.00%	67	57.76%	0.57	0.451	0.097
	Positive result	6	60.00%	49	42.24%			
	Total	10	100.00%	116	100.00%			
Clinical Department of Otolaryngology	Negative result	2	100.00%	7	70.00%	0.80	1.000	0.258
	Positive result	0	0.00%	3	30.00%			
	Total	2	100.00%	10	100.00%			
Clinical Department of Pediatrics	Negative result	2	100.00%	28	84.85%	0.01	1.000	0.101
	Positive result	0	0.00%	5	15.15%			
	Total	2	100.00%	33	100.00%			
Clinical Department of Internal Medicine I	Negative result	1	20.00%	88	61.54%	1.96	0.161	0.153
	Positive result	4	80.00%	55	38.46%			
	Total	5	100.00%	143	100.00%			
Department of Trauma and Orthopaedic Surgery	Negative result	0	0.00%	36	50.70%	0.01	1.000	0.119
	Positive result	1	100.00%	35	49.30%			
	Total	1	100.00%	71	100.00%			
Department of Hematology	Negative result	9	69.23%	117	73.13%	0.01	1.000	0.023
	Positive result	4	30.77%	43	26.88%			
	Total	13	100.00%	160	100.00%			
Department of Cardiac Surgery	Negative result	9	75.00%	14	77.78%	0.01	1.000	0.032
	Positive result	3	25.00%	4	22.22%			
	Total	12	100.00%	18	100.00%			
Cardiology Department	Negative result	1	100.00%	49	57.65%	0.01	1.000	0.092
	Positive result	0	0.00%	36	42.35%			
	Total	1	100.00%	85	100.00%			
Department of Pulmonology and Pulmonary Oncology	Negative result	1	100.00%	85	64.89%	0.01	1.000	0.064
	Positive result	0	0.00%	46	35.11%			
	Total	1	100.00%	131	100.00%			
Clinical Department of Rehabilitation	Negative result	4	25.00%	10	26.32%	0.01	0.920	0.014
	Positive result	12	75.00%	28	73.68%			
	Total	16	100.00%	38	100.00%			
Department of Rheumatology	Negative result	0	0.00%	48	78.69%	0.44	0.509	0.237
	Positive result	1	100.00%	13	21.31%			
	Total	1	100.00%	61	100.00%			
Internal Department II	Negative result	65	51.59%	161	69.40%	11.13	0.001	0.176
	Positive result	61	48.41%	71	30.60%			
	Total	126	100.00%	232	100.00%			
Intensive Care Unit	Negative result	612	69.15%	37	59.68%	2.41	0.120	0.050
	Positive result	273	30.85%	25	40.32%			
	Total	885	100.00%	62	100.00%			
Hospital Clinical Department of Neurosurgery	Negative result	23	44.23%	23	52.27%	0.62	0.432	0.080
	Positive result	29	55.77%	21	47.73%			
	Total	52	100.00%	44	100.00%			
